# Characterization of Intact Proviruses in Blood and Lymph Node from HIV-Infected Individuals Undergoing Analytical Treatment Interruption

**DOI:** 10.1128/JVI.01920-18

**Published:** 2019-04-03

**Authors:** Line K. Vibholm, Julio C. C. Lorenzi, Joy A. Pai, Yehuda Z. Cohen, Thiago Y. Oliveira, John P. Barton, Marco Garcia Noceda, Ching-Lan Lu, Yuria Ablanedo-Terrazas, Perla M. Del Rio Estrada, Gustavo Reyes-Teran, Martin Tolstrup, Paul W. Denton, Tine Damsgaard, Ole S. Søgaard, Michel C. Nussenzweig

**Affiliations:** a Department of Clinical Medicine, Aarhus University, Aarhus, Denmark; b Department of Infectious Diseases, Aarhus University Hospital, Aarhus, Denmark; c Laboratory of Molecular Immunology, The Rockefeller University, New York, New York, USA; d Department of Physics and Astronomy, University of California, Riverside, California, USA; e Center for Research in Infectious Diseases, National Institute of Respiratory Diseases, Mexico City, Mexico; f Howard Hughes Medical Institute, The Rockefeller University, New York, New York, USA; Emory University

**Keywords:** ATI, HIV-1, infectious diseases, recombination, lymph node

## Abstract

The role of lymphoid tissue as a potential source of HIV-1 rebound following interruption of antiretroviral therapy (ART) is uncertain. To address this issue, we compared the latent viruses obtained from CD4^+^ T cells in peripheral blood and lymph nodes to viruses emerging during treatment interruption. Latent viruses were characterized by sequencing near-full-length (NFL) proviral DNA and *env* from viral outgrowth assays (VOAs). Five HIV-1-infected individuals on ART were studied, four of whom participated in a clinical trial of a TLR9 agonist that included an analytical treatment interruption. We found that 98% of intact or replication-competent clonal sequences overlapped between blood and lymph node. In contrast, there was no overlap between 205 latent reservoir and 125 rebound sequences in the four individuals who underwent treatment interruption. However, rebound viruses could be accounted for by recombination. The data suggest that CD4^+^ T cells carrying latent viruses circulate between blood and lymphoid tissues in individuals on ART and support the idea that recombination may play a role in the emergence of rebound viremia.

**IMPORTANCE** HIV-1 persists as a latent infection in CD4^+^ T cells that can be found in lymphoid tissues in infected individuals during ART. However, the importance of this tissue reservoir and its contribution to viral rebound upon ART interruption are not clear. In this study, we sought to compare latent HIV-1 from blood and lymph node CD4^+^ T cells from five HIV-1-infected individuals. Further, we analyzed the contribution of lymph node viruses to viral rebound. We observed that the frequencies of intact proviruses were the same in blood and lymph node. Moreover, expanded clones of T cells bearing identical proviruses were found in blood and lymph node. These latent reservoir sequences did not appear to be the direct origin of rebound virus. Instead, latent proviruses were found to contribute to the rebound compartment by recombination.

## Abstract (precis)

HIV-1 persists as a latent infection in CD4^+^ T cells that can be found in lymphoid tissues in infected individuals during ART. However, the importance of this tissue reservoir and its contribution to viral rebound upon ART interruption are not clear. In this study, we sought to compare latent HIV-1 from blood and lymph node CD4^+^ T cells from five HIV-1-infected individuals. Further, we analyzed the contribution of lymph node viruses to viral rebound. We observed that the frequencies of intact proviruses were the same in blood and lymph node. Moreover, expanded clones of T cells bearing identical proviruses were found in blood and lymph node. These latent reservoir sequences did not appear to be the direct origin of rebound virus. Instead, latent proviruses were found to contribute to the rebound compartment by recombination.

## INTRODUCTION

Antiretroviral therapy (ART) is an effective treatment for HIV-1 infection. However, HIV-1 persists as proviral DNA in CD4^+^ T cells and represents a latent reservoir that demonstrates remarkable stability (1–3). Upon ART interruption, nearly all individuals experience viral rebound within 1 to 6 weeks (4–6). Nevertheless, most integrated proviruses are defective (7, 8), and only a small percentage can replicate and produce infectious virions. Latent viruses can be recovered from the reservoir by viral outgrowth assays (VOAs) and by PCR amplification of integrated proviruses (9, 10). By using these methods, latent viruses have been recovered from peripheral blood, lymph nodes, gut-associated lymphoid tissue (GALT), spleen, central nervous system (CNS), liver, lungs, kidney, adipose tissue, and genital tract (11–17).

In four recent studies, comparisons of circulating latent viruses and rebound viruses showed a very limited number of overlapping *env* sequences (10, 18–20). One potential explanation for this observation is that latent viruses found in CD4^+^ T cells in lymphoid and other tissues differ from those in circulation and that these sequestered cells are responsible for the rebound viremia. To examine the relationship between latent viruses in circulation and in lymph node, we studied five individuals who had concurrent blood draws and lymph node biopsies. Four of these individuals were enrolled in a clinical trial that included an analytical treatment interruption (ATI) after 24 weeks of therapy with a TLR9 agonist.

This article was submitted to an online preprint archive (21).

## RESULTS

### Intact and defective latent viruses from blood and lymph node CD4^+^ T cells overlap.

To investigate latent viruses from lymph node and blood CD4^+^ T cells, we obtained mononuclear cells from lymph nodes (LNMCs) and peripheral blood (PBMCs) from five HIV-1-infected individuals who had been virally suppressed on ART for a median of 7.3 years (range, 1.8 to 13.4 years) (Table 1). Four participated in an interventional trial in which a TLR9 agonist was coadministered with ART for 24 weeks, followed by ART interruption (ClinicalTrials.gov registration no. NCT02443935) (22). For these individuals, PBMCs and LNMCs were collected in the last 2 weeks of the 24-week period, before ART interruption. The aim of adjunctive TLR9 agonist treatment was to improve antiviral immunity in general and HIV-specific immunity in particular (22, 23). We also obtained concurrent PBMCs and LNMCs from a fifth HIV-1-infected individual (designated LFSO) who had been suppressed on ART for 1.3 years and who did not receive any investigational therapy. To obtain full-length *env* sequences from viruses that were replication competent and/or genetically intact, CD4^+^ T cells from blood and lymph node were assayed by VOA and near-full-length (NFL) sequencing (10).

**TABLE 1:** Patient characteristics

Baseline characteristic (*n* = 5)	Value
Gender male, no. (%)	5 (100)
Race or ethnicity, no. (%)	
Caucasian	4 (80)
Hispanic	1 (20)
Age (yrs), median (range)	51 (28–57)
Yrs since HIV-1 diagnosis, median (range)	9.3 (2–31)
Yrs from HIV diagnosis to ART initiation, median (range)	0.25 (0–18)
Yrs on ART, median (range)	7.3 (1.8–13.4)
ART regimen, no. (%)	
Protease inhibitor based	2 (40)
NNRTI based^*a*^	3 (60)
Nadir CD4^+^ T cell count (cells/mm^3^), median (range)	390 (210–690)

a NNRTI, nonnucleoside reverse transcriptase inhibitors.

**FIG 1:**
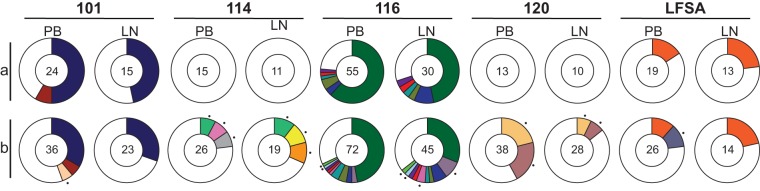
Sequence identity between peripheral blood (PB) and lymph node (LN). (a) Pie charts showing the distribution of intact near-full-length (NFL) and VOA-derived *env* sequences. Numbers in the center of the circles represent the total numbers of intact/replication-competent sequences obtained. White areas in the pie charts represent sequences obtained once (singles). Colored areas represent sequences obtained more than once (clones). Clones, which are found in both PB and LN within an individual, share the same color between the two PB/LN pie charts. The size of the slices in the pie charts is proportional to the relative size of the clone. There is no significant difference between the frequencies of clones in PB and LN for any participant (two-sided Fisher’s exact test). (b) Pie charts showing the distribution of combined intact and defective *env* sequences from NFL and VOA-derived *env* sequences. Small black circles denote defective clones. There is no significant difference between the frequencies of clones in PB and LN for any participant (two-sided Fisher’s exact test).

We obtained 205 latent virus sequences: 93 and 79 intact NFL sequences from blood and lymph node, respectively, and 33 from VOA sequences from blood. Phylogenetic analysis showed that each participant analyzed was infected with genetically distinct clade B viruses (extended data set). Sequence analysis revealed that 44% of all sequences belonged to expanded clones, 98% of which overlapped between blood and lymph node (i.e. identical sequences) (Fig. 1a). For example, in participant 116, the 6 distinct clones found in PBMCs were also present in LNMCs at similar frequencies. Among the 9 clones found in 3 participants, there was only a single instance where a clone was found in one compartment but not the other (participant 101). In that participant, a small clone that appeared in PBMCs was not found in LNMCs, from which fewer sequences were obtained due to sample availability. Clones were absent in participants 114 and 120, where sample availability was limited and latent viruses were diverse.

To determine whether defective viruses were also similar between blood and lymph node, we combined intact and defective *env* sequences from all NFL sequencing and VOAs (327 sequences). Overall, 43% of all *env* sequences belonged to expanded clones (143 sequences), and 89% of the clones overlapped between blood and lymph node (Fig. 1b). In participants 114 and 120, in whom no replication-competent and/or genetically intact clones were found, a total of 5 defective clones were present, 3 of which were found in both LNMCs and PBMCs. We conclude that CD4^+^ T cells in peripheral blood and lymph node contain overlapping sets of proviruses.

**FIG 2:**
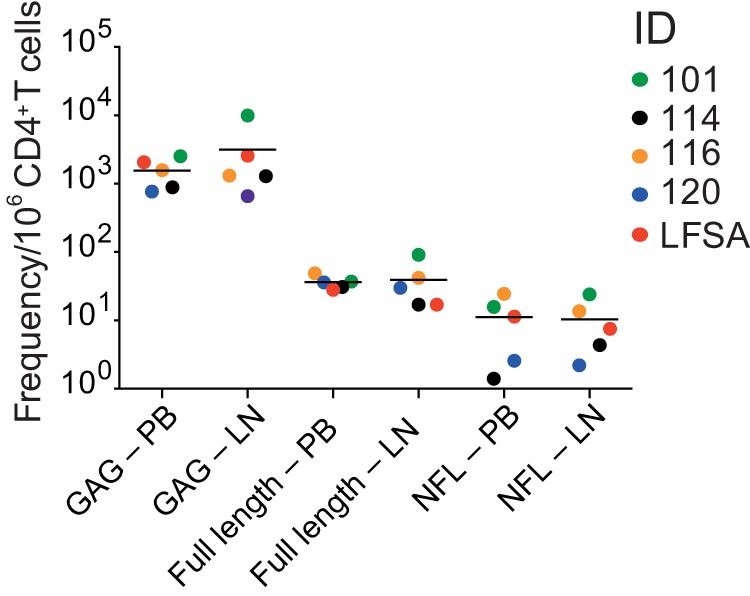
Quantitative analysis of the latent reservoir. Frequency of *gag*^+^ cells per 10^6^ CD4^+^ T cells in LN and PB, frequency of full-length viruses (amplicon size determined using 0.8% agarose gel; i.e., at least one combination of either A+C, A+D, B+C, or B+D [9]) per 10^6^ CD4^+^ T cells in LN and PB, and frequency of intact near-full-length (NFL) sequences per 10^6^ CD4^+^ T cells in LN and PB are shown. There was no statistical difference between the frequencies of *gag*^+^ cells (*P* value = 0.31), full-length viruses (*P* value = 0.63), or intact NFL sequences (*P* value = 0.81) per 10^6^ CD4^+^ T cells (Wilcoxon matched-pairs signed-rank test).

To further examine the relative proviral nucleic acid content in peripheral blood and lymph node, we compared the frequencies of (i) *gag*^+^ cells, (ii) full-length genomes, and (iii) intact NFL proviruses. In all cases, there were no statistically significant differences in relative proviral nucleic acid content between peripheral blood and lymph node (Fig. 2). Thus, in our cohort, the frequencies of intact and defective HIV-1 proviruses in CD4^+^ T cells were similar in peripheral blood and lymph node.

### Relationship between plasma rebound viruses and reservoir viruses.

**FIG 3:**
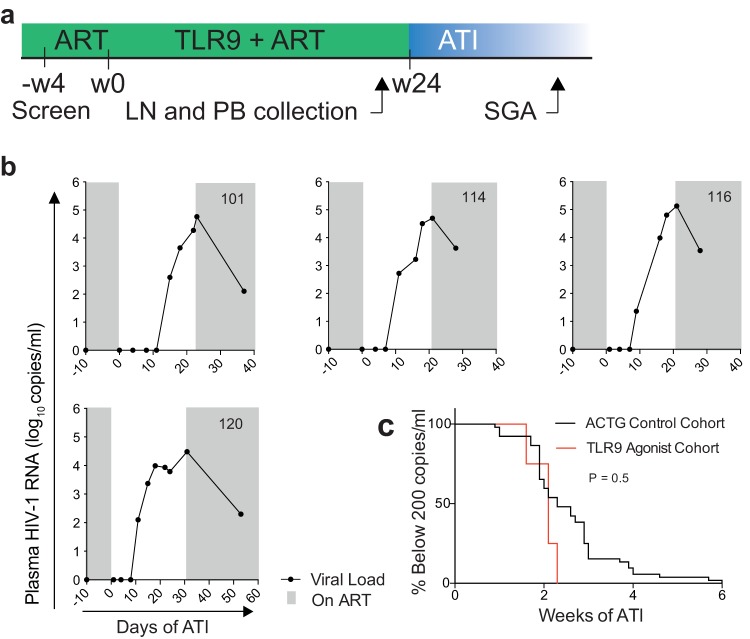
TLR9 agonist study design and rebound analysis. (a) Study design. The green area represents the time on ART before enrollment and during the 24 weeks of TLR9 agonist treatment. The blue area represents the time off ART. Weeks (w) elapsed are shown. Lymph node (LN) and peripheral blood (PB) were collected 1 to 2 weeks before initiation of the analytical treatment interruption (ATI). Single-genome analysis (SGA) was performed on plasma from the time of viral rebound. (b) Plasma HIV-1 RNA levels (left *y* axis) and days elapsed following ATI (*x* axis). The lower limit of HIV-1 RNA detection was 20 copies/ml. Gray shaded areas depict time on ART. (c) Kaplan-Meier plot summarizing the time to rebound for the four TLR9 agonist trial participants (red line) compared to a cohort of 52 ACTG trial participants (black line) who underwent ATI without intervention. The log rank test *P* value applies to the comparison of time to rebound for the TLR9 agonist-treated cohort and that for the ACTG cohort.

**FIG 4:**
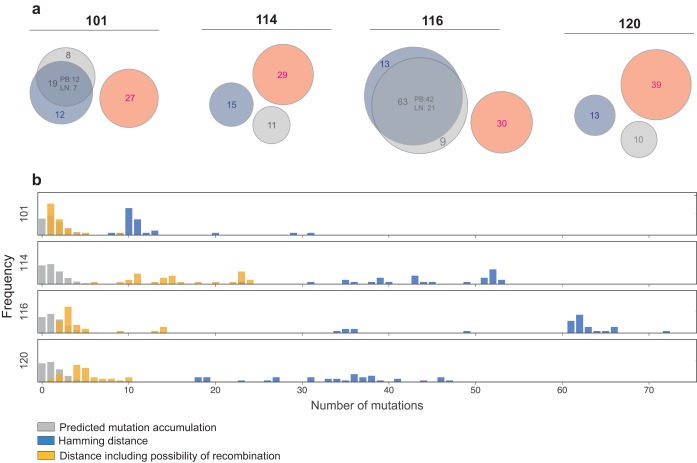
Sequence identity between peripheral blood (PB), lymph node (LN), and rebound single-genome assay (SGA) viruses. (a) Venn diagrams depicting *env* sequences from PB near-full-length (NFL) and PB VOA (blue) sequences, LN NFL (gray) sequences, and SGA sequences from the time of viral rebound (pink). The numbers of sequences obtained are indicated in the circles. The relative size of the overlapping areas is proportional to the number of identical sequences. (b) The *y* axis on the histograms shows the frequency of *env* sequences, and the *x* axis shows the nucleotide distance in number of mutations. The gray bars represent the expected distance between the latent reservoir and rebound viruses based on a simulation of the accumulation of mutations for each participant during the ATI. The blue bars represent the observed Hamming distance found between latent reservoir viruses and rebound viruses, ignoring indels. The yellow bars represent the observed distance between the latent reservoir viruses and rebound viruses when the possibility of recombination is included in the analysis.

The four participants enrolled in the TLR9 trial underwent treatment interruption at week 24 (Fig. 3a). The time from ART withdrawal to viral rebound was between 9 and 15 days (Fig. 3b), which is not significantly different from that of a noninterventional ATI control cohort of 52 participants (ACTG cohort) (24–26) (Fig. 3c) (*P* = 0.5, log rank test). One hundred twenty-five full-length *env* sequences were obtained by single-genome analysis (SGA) from rebound plasma, none of which overlapped with 173 replication-competent and/or genetically intact latent reservoir sequences (Fig. 4a). To determine whether mutations accumulating during rebound could account for the divergence between the latent reservoir and rebound viruses, we used a stochastic mutation simulation model (18, 27, 28). We found no instance in which rebound sequences could be accounted for by mutation alone (Fig. 4b).

**FIG 5:**
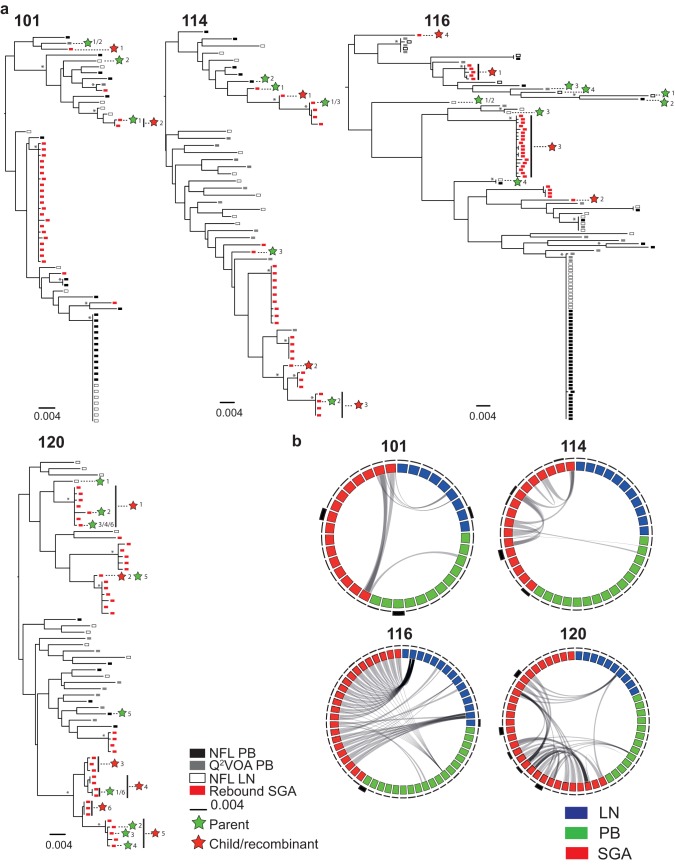
Comparison of *env* from intact sequences obtained from lymph node (LN), peripheral blood (PB) cells, and rebound viruses from participants 101, 114, 116, and 120. (a) Maximum likelihood phylogenetic trees of *env* from near-full-length (NFL) LN and PB sequences, viral outgrowth assay (VOA) PB culture sequences, and plasma single-genome analysis (SGA) sequences. Symbols are defined in the graph key. Asterisks indicate nodes with significant bootstrap values (bootstrap support ≥ 90%). Green stars indicate parent sequences, which undergo recombination to produce a child sequence (red star). Each recombination event has a number (indicated next to the colored star). (b) Circos plots showing the connection between the two parent sequences and the child/recombinant sequence, which are also depicted in the trees. Blue blocks represent latent reservoir LN sequences. Green blocks represent latent reservoir PB sequences. Red blocks represent plasma virus sequences. Clonal sequences are depicted once. The thickness of the outer black lines surrounding the circles represents the number of sequences retained within the clone, i.e., thin black lines are single sequences, and thicker lines represent sequences obtained several times. Gray lines inside the Circos plots indicate the recombination event. The parent/child relationship is shown in the trees.

To determine whether recombination between latent blood and lymph node viruses could account for the rebound viruses, we analyzed intact NFL and VOA *env* sequences using the 3SEQ recombination algorithm (http://mol.ax/software/ 3seq /). Rebound viruses in all four individuals showed evidence of recombination (Fig. 5a and b, extended data set). There were no instances in which a parent latent virus involved in a recombination event was an expanded clone. In two of the four participants, recombination was observed between latent viruses that derived from PBMCs and LNMCs. By including the possibility of mutation and recombination, we were able to account for 53% of the rebound sequences. We conclude that latent viruses present in both lymph node and peripheral blood may contribute to rebound viremia through recombination.

## DISCUSSION

HIV-1 proviruses are present in all tissues analyzed to date (11–17). However, the relationship between viruses found at different sites and their contribution to persistence are not well defined. Our data suggest that the latent viruses found in circulation overlap with those found in lymph nodes and that the most prevalent viruses in these compartments are not typically found among rebound viruses during treatment interruption.

Four of five individuals analyzed received up to 24 weeks of TLR9 agonist treatment prior to sample collection. The aim of adjunctive TLR9 agonist treatment was to improve antiviral immunity in general and HIV-specific immunity in particular (22). The participants we analyzed who received TLR9 treatment did not demonstrate delayed time to viral rebound, and the dynamics of plasma HIV-1 RNA during rebound was similar to that of historical controls. We therefore consider it unlikely that the TLR9 agonist treatment in these individuals significantly impacted the composition or distribution of latent viruses or affected rebound viremia.

It has been suggested that lymph nodes serve as a sanctuary site for latent HIV-1 (29, 30). However, we found the same overall frequencies of HIV-1 proviruses in blood and lymph node CD4^+^ T cells. Moreover, the two compartments contained similar numbers of genetically intact viruses and similar frequencies of the same clonal latent viruses. Thus, expanded CD4^+^ T cell clones bearing latent viruses circulate between these two compartments. Finally, rebound viruses did not overlap with either blood or lymph node but instead appeared to represent recombinants.

Among 205 latent virus sequences derived from LNMCs and PBMCs from five individuals, 44% were expanded clones, similar to although slightly lower than the frequency of clones in previous reports (18, 31–34). In participant 116, who had largest number of cells available for analysis, 3 of 6 clones, including the largest clone, were sequenced from both proviral DNA and VOA sequences. Thus, these clonal viruses were shown to be present at high frequency in LNMCs and PBMCs and capable of replication. Nevertheless, these viruses did not emerge during rebound viremia. Additionally, these viruses did not serve as parents in recombination events. This is consistent with findings in previous studies that expanded latent clones only very rarely serve as parents in the recombination events that give rise to rebound viruses (10, 18, 19). Thus, the importance of these highly expanded clones for the persistence of the HIV reservoir and the emergence of rebound viremia remains uncertain.

Our findings are consistent with those of other reports that failed to find evidence of compartmentalization among latent viruses in blood and tissues (35, 36). There were no demonstrable differences between levels of *gag*^+^ proviral DNA, full-length genomes, or intact NFL DNA in LNMCs and PBMCs. Of note, the levels of HIV DNA in GALT have been found to be ∼2.5-fold higher than that in PBMCs (35). Thus, the frequency of proviral DNA appears to differ between GALT and peripheral lymph nodes, and it remains possible that the cellular source of rebound viremia is restricted to specific groups of lymphoid tissues such as GALT. Moreover, our work is limited by the numbers of individuals and CD4^+^ T cells analyzed, and therefore we cannot rule out the possibility that a subset of lymph node cells, such as CD4^+^ T_FH_ cells, are enriched in or harbor a specific group of latent viruses (37).

Three other studies have also shown little or no overlap between circulating latent viruses and rebound viruses (10, 18, 19). Including the data reported here, there are only 3 overlapping sequences among 1,816 independently derived latent reservoir viruses and 642 rebound viruses. Instead, the latent and rebound viruses appear to be related by recombination. Interestingly, this study is the first to document instances in which the two parent latent viruses in a recombination event derived from different anatomical compartments. This finding reinforces the notion that latently infected CD4^+^ T cells circulate between lymph nodes and peripheral blood.

There are a number of potential explanations for the lack of concordance between latent and rebound viruses. One possibility is that latent reservoir sampling has been inadequate. For example, the active reservoir responsible for rebound might be found primarily in a tissue that has not been assayed. A second nonexclusive possibility is that the majority of the latent viruses in blood and lymph node fail to emerge *in vivo* because they are in some way unfit to do so. For example, the majority of latent viruses assayed could be susceptible to immune pressure, resulting in selection of a subset of rebound viruses which can escape antiviral immunity *in vivo*, possibly by recombination (38, 39). While there was a high degree of recombination found within rebound virus *env* sequences, the presence and degree of recombination in other genes remain to be determined. Further analysis of latent proviral sequences may provide insights into the mechanisms that account for the observed discordance between latent and rebound viruses.

In conclusion, the data reported indicate that the majority of the latent clonal viruses found in blood are also found in the lymph nodes and add to the growing body of literature suggesting that rebound viruses are either not present in or are rare components of the latent reservoir found in circulation.

## MATERIALS AND METHODS

### Study design and participants.

This study was approved by the Danish Research Health Ethics Committee (1-10-72-133-17) and the Danish Data Protection Agency, by The Rockefeller University Institutional Review Board (protocol TSC-0910) and Research Committee, and the Research Ethics Committee of the Instituto Nacional de Enfermedades Respiratorias Ismael Cosío Villegas. Samples for this study were generated from a clinical trial conducted in 2016 to 2017 at Aarhus University Hospital, Aarhus, Denmark (22) (ClinicalTrials.gov identifier NCT02443935). The clinical study was approved by the Danish Research Health Ethics Committee (case no. 1-10-72-10-15), the Danish Medicines Agency (2015014125), and the Danish Data Protection Agency. Participants were recruited from the Department of Infectious Diseases Outpatient Clinic at Aarhus University Hospital and signed a written informed consent before any study procedures. Inclusion criteria were as follows: plasma HIV-1 RNA of <50 copies/ml, CD4^+^ T cell count of >350 cells/μl, age of >18 years, on combination antiretroviral therapy (cART) for >12 months, and ability to provide informed consent (a complete list of inclusion and exclusion criteria is accessible at ClinicalTrials.gov). Inclusion criteria for participation in the ATI were as follows: HIV RNA of <20 copies/ml, CD4^+^ T cell count of >350 cells/μl, and written informed consent to withdrawal of cART. The Cobas TaqMan HIV-1 test (version 2.0 by Roche) was used to assess plasma viral load (pVL) twice a week, and the CD4^+^ T cell count was measured every other week. Participants were called in for reinitiation of ART and collection of plasma after two measurements of HIV-1 RNA of >5,000 copies/ml or a CD4^+^ T cell count of <350 cells/μl. Data on the historical control cohort (ACTG trials 371 [24], A5024 [25], A5068 [26], and A5197 [44]) were used for comparison with viral rebound data from the four participants in this study. The studies were carried out as an ATI without any further interventions and included 52 participants. Inclusion criteria for the ACTG cohort were as follows: age of 18 to 65 years, on cART for >12 months, plasma HIV-1 RNA level of <50 copies/ml for at least 12 months before ATI initiation, a CD4^+^ T cell count at time of ATI initiation of >500 cells/μl, and a nadir CD4^+^ T cell count of >200 cells/μl. Viral load was measured weekly until viral rebound occurred.

### VOA.

The viral outgrowth assay (VOA) was performed using PBMCs from peripheral blood (PB) as previously described (Q^2^VOA) (34). Briefly, PBMCs were isolated by density centrifugation on Ficoll (Thermo Scientific). CD4^+^ T cells were isolated from the cryopreserved PBMCs through negative selection with magnetic beads (Miltenyi). Purified CD4^+^ T cells (0.1 × 10^6^) were cultured with 0.2 × 10^6^ irradiated heterologous PBMCs from an HIV-1-negative donor in 200 μl medium (RPMI 1640 [Gibco] supplemented with 10% fetal bovine serum [FBS] [HyClone; Thermo Scientific]), 1% penicillin/streptomycin (Gibco), 1 μg/ml phytohemagglutinin (Life Technologies), and 100 U/ml interleukin 2 (IL-2; Peprotech) at 37°C and 5% CO_2_. At this density, less than 30% of the cultures became p24 positive. Cultures were rested overnight, and after 24 h, 125 μl of medium was discarded and 10^4^ MOLT4-CCR5 cells were added to each well as target cells. At day 5, 100 μl of medium was replaced with fresh medium. At day 14, the supernatant of each well was tested for p24 production using ELISA as previously described (40).

### VOA sequence amplification.

Extraction of RNA and generation of cDNA and amplification of full-length *env* were performed as previously described (18, 34). The 1% 96-well E-Gels (Invitrogen) were used to visualize amplified *env* PCR products and select the bands with the expected HIV-1 envelope size. Selected PCR products were subjected to library preparation using a Nextera DNA sample preparation kit (Illumina) as previously described (35). Briefly, DNA was diluted in nuclease-free water to 10 to 20 ng per well and subjected to tagmentation. The Illumina Nextera index kit was then used to ligate tagmented DNA to barcoded sequencing adapters. Subsequently, AmPure XP Beads (Agencourt) were used to purify DNA. Each library consisted of 96 different samples, which were subjected to paired-end sequencing using Illumina MiSeq Nano 300 (Illumina) cycle kits at a final concentration of 12 pM.

### SGA of plasma rebound virus.

Single-genome amplification (SGA) and sequencing of HIV-1 *env* genes were performed as previously described (41–43).

### Near-full-length genome amplification.

CD4^+^ T cells were isolated from cryopreserved PBMCs and lymph node mononuclear cells (LNMCs) using magnetic beads (Miltenyi). For genomic DNA extraction, we used a Gentra Puregene cell kit (Qiagen), according to the manufacturer’s instructions. Near-full-length (NFL) sequencing was done by an initial limiting-dilution seminested PCR amplifying the *gag* gene using the primers 3GagIN, 5GAGIN, and 3GAGININ. If these primers failed, we used the primers GAGB5out, GAGB3out, GAGB5in, and GAGB3in as previously described (7, 9). *gag* PCR products were visualized using 1% 96-well E-Gels (Invitrogen). Dilutions with <30% positive PCR wells were selected for further analysis. According to Poisson statistics, this dilution has >90% probability of containing one HIV-1 DNA molecule in each PCR. The NFL HIV-1 genome was amplified as a nested PCR with primers and cycling conditions as previously described (7, 9). Briefly, the outer 9,064-bp PCR was performed using the primers BLOuterF and BLOuterR and High Fidelity Platinum *Taq* polymerase (Invitrogen). For the nested PCR, 0.75 μl was transferred and the *env* gene was amplified using the primers envB5out and envB3out. Wells containing an intact *env* gene were selected using 1% 96-well E-Gels (Invitrogen), and the corresponding outer PCR products were collected for further analyses. NFL outer PCR products were subjected to a nested PCR to generate four segments, A, B, C, and D, comprising overlapping parts of the genome. The PCR products were visualized on a 0.8% agarose gel to determine amplicon size. PCR products with the accepted size (A, 4,449 bp; B, 5,793 bp; C, 6,385 bp; D, 4,778 bp) were combined as either A+C, A+D, B+C, or B+D and subsequently subjected to library preparation and sequencing using Illumina MiSeq Nano 300 (Illumina) cycle kits at a final concentration of 12 pM, as described above. Assembly and analysis of HIV-1 genome sequencing were performed as previously described (10).

### Identification of intact proviruses and construction of phylogenetic trees.

To identify intact NFL sequences, we aligned assembled sequences to HXB2. This allowed us to identify premature stop codons, out-of-frame insertions or deletions (indel), or packaging signal (Ψ) deletions and mutations using custom Python scripts. Intact genomes were identified as sequences containing productive genes and the major splice donor (MSD) site. Sequences which had a deleted or mutated MSD site were categorized as Ψ-MSD deletion/mutation. The Los Alamos HIV Sequence Database Hypermut tool was used to determine the presence of APOBEC-induced G-to-A hypermutation in the remaining NFL sequences. Sequences which were not categorized as hypermutated were considered defective due to indels/nonsense mutations. Trees combining all sequences for each individual (intact and defectives) are shown in the supplemental material (extended data set).

Maximum likelihood phylogenetic trees were constructed as previously described (10). To assess for cross-contamination of samples, we also generated a neighbor-joining (NJ) tree, which included all sequences obtained for the entire analyses and hereby confirmed that all sequences clustered correctly. Maximum likelihood phylogenetic trees showing all sequence names are in the supplemental material.

### Recombination analysis of *env* sequences.

Multiple alignment of nucleotide sequences and the recombination analysis were performed as previously described (10, 18). Briefly, *env* sequences from NFL, VOA, and SGA rebound sequences were analyzed for occurrence of recombination by the 3SEQ recombination algorithm (http://mol.ax/software/ 3seq /). Sequences with statistical evidence of recombination (i.e., rejection of the null hypothesis of clonal evolution) are represented in a Circos plot (http://circos.ca/).

### Accession number(s).

Sequence data generated in this study have been submitted to GenBank under accession numbers MK169416 to MK170053.

## Supplementary Material

Supplemental file 1
