# Supplementary material for: Characterization of Intact Proviruses in Blood and Lymph Node from HIV-Infected Individuals Undergoing Analytical Treatment Interruption
Source: J Virol. 2019 Apr 3;93(8):e01920-18. doi: 10.1128/JVI.01920-18 (PMC6450127; doi:10.1128/JVI.01920-18)

Extended Dataset Figure 1

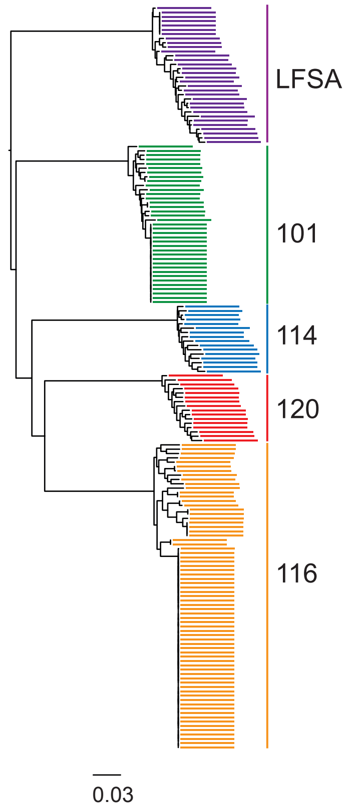

Extended Data Figure 1. Phylogenetic tree depicting intact sequences from all five participants. Maximum likelihood phylogenetic tree showing all env sequences from intact near full length sequences, viral outgrowth assay sequences and rebound SGA sequences from all 5 participants. Each participant is illustrated in a different color. The tree is constructed using RAxML v.8.0.22 with a GTR GAMMA substitution model with 1000 bootstrap replicates and is midpoint rooted.

Extended Dataset Figure 2

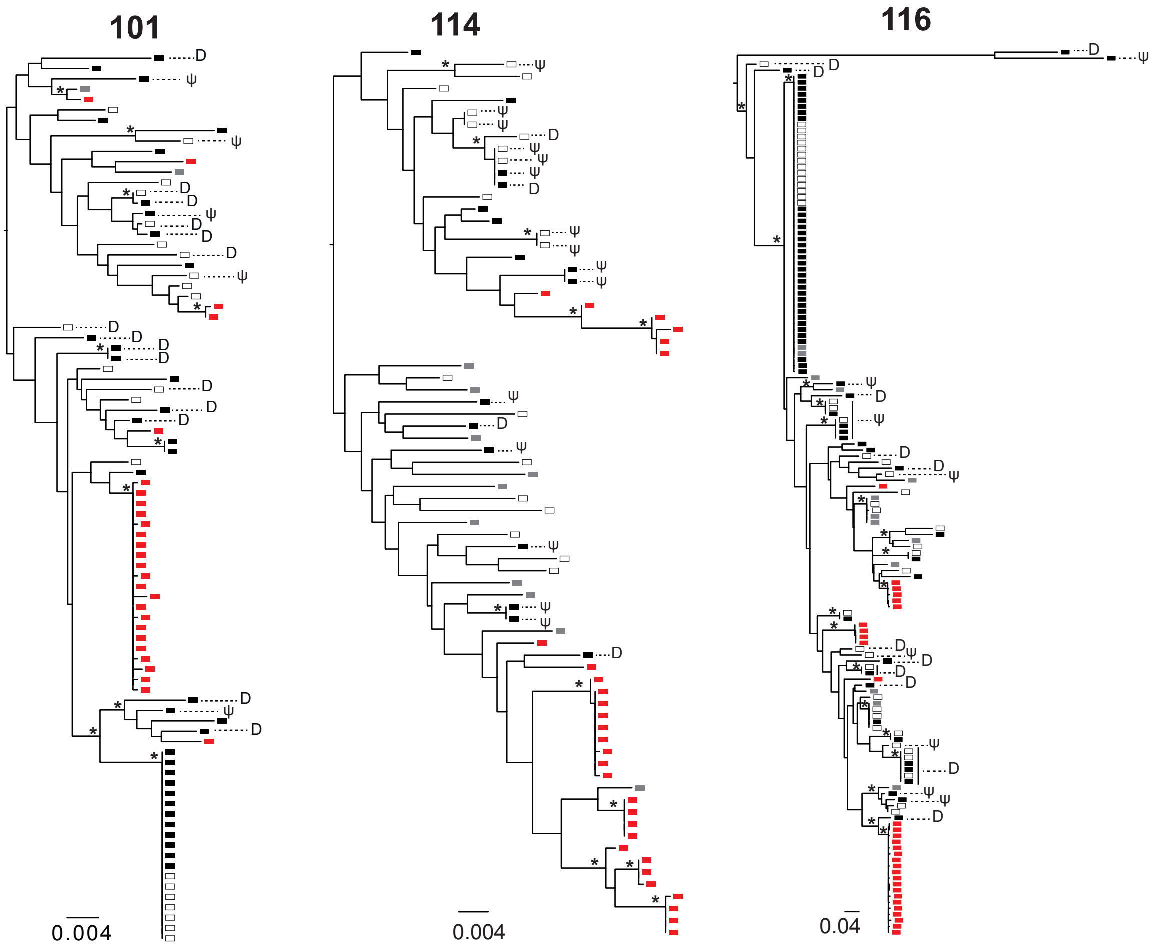

Extended Data Figure 2. Phylogenetic trees showing all env sequences. Maximum likelihood phylogenetic trees with env sequences from intact near full-length (NFL), defective NFL, viral outgrowth assay (VOA) and single genome amplification (SGA). The tree is midpoint rooted. Open rectangles denote NFL sequences from lymph node (LN) CD4+ T cells. Closed black rectangles represent sequences 1 from NFL peripheral blood (PB). Closed grey rectangles are VOA culture sequences and red rectangles are SGA. NFL Sequences with packaging signal mutations are marked with ”ψ”. All other defects in NFL are marked as ”D”. Asterisks indicate nodes with significant bootstrap values (bootstrap support ≥ 90%).

Extended Dataset Figure 2 (continued)

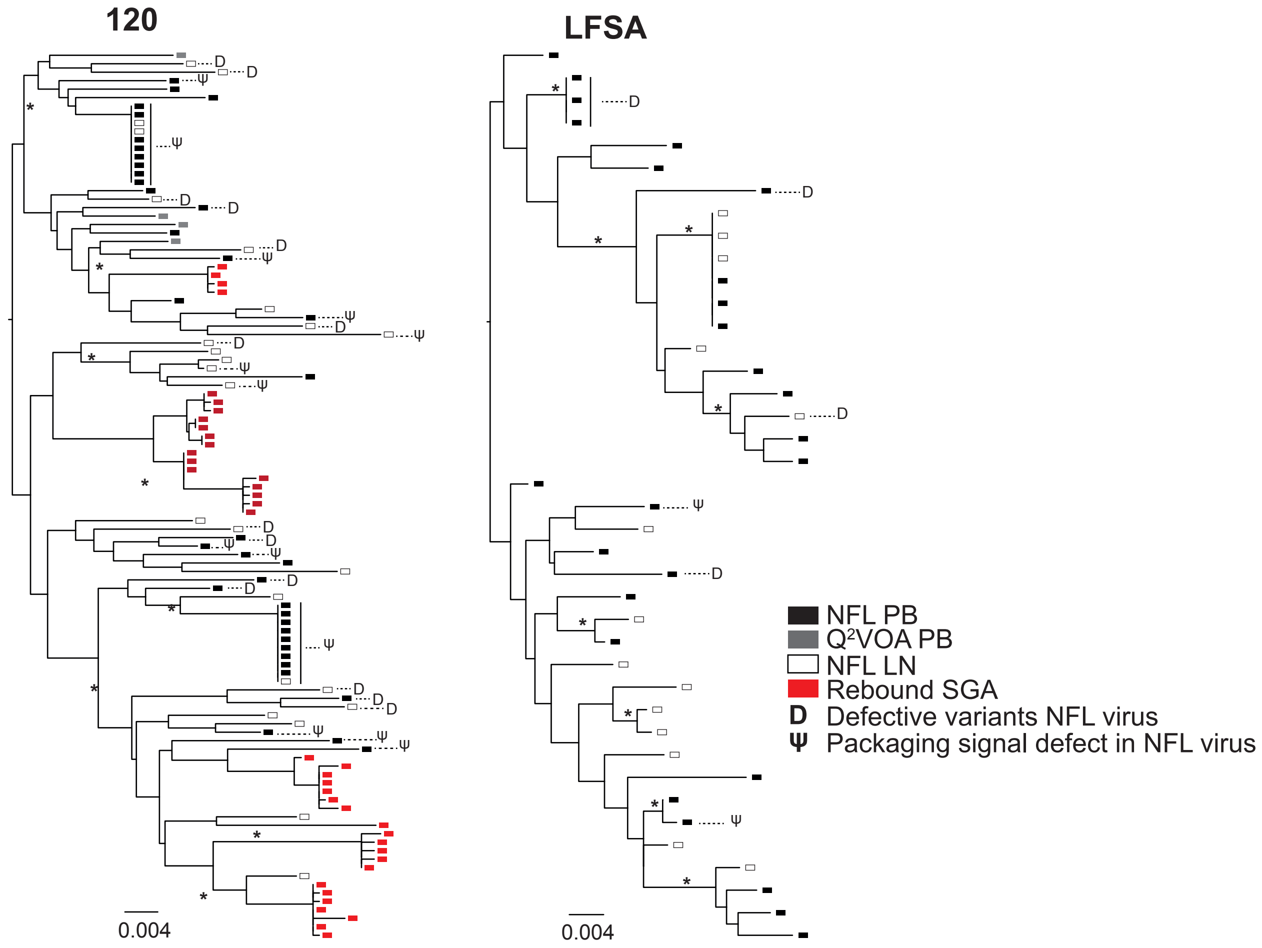

Extended Data Figure 2. Phylogenetic trees showing all env sequences. Maximum likelihood phylogenetic trees with env sequences from intact near full-length (NFL), defective NFL, viral outgrowth assay (VOA) and single genome amplification (SGA). The tree is midpoint rooted. Open rectangles denote NFL sequences from lymph node (LN) CD4+ T cells. Closed black rectangles represent sequences 1 from NFL peripheral blood (PB). Closed grey rectangles are VOA culture sequences and red rectangles are SGA. NFL Sequences with packaging signal mutations are marked with ”ψ”. All other defects in NFL are marked as ”D”. Asterisks indicate nodes with significant bootstrap values (bootstrap support ≥ 90%).

Extended Data Figure 3.

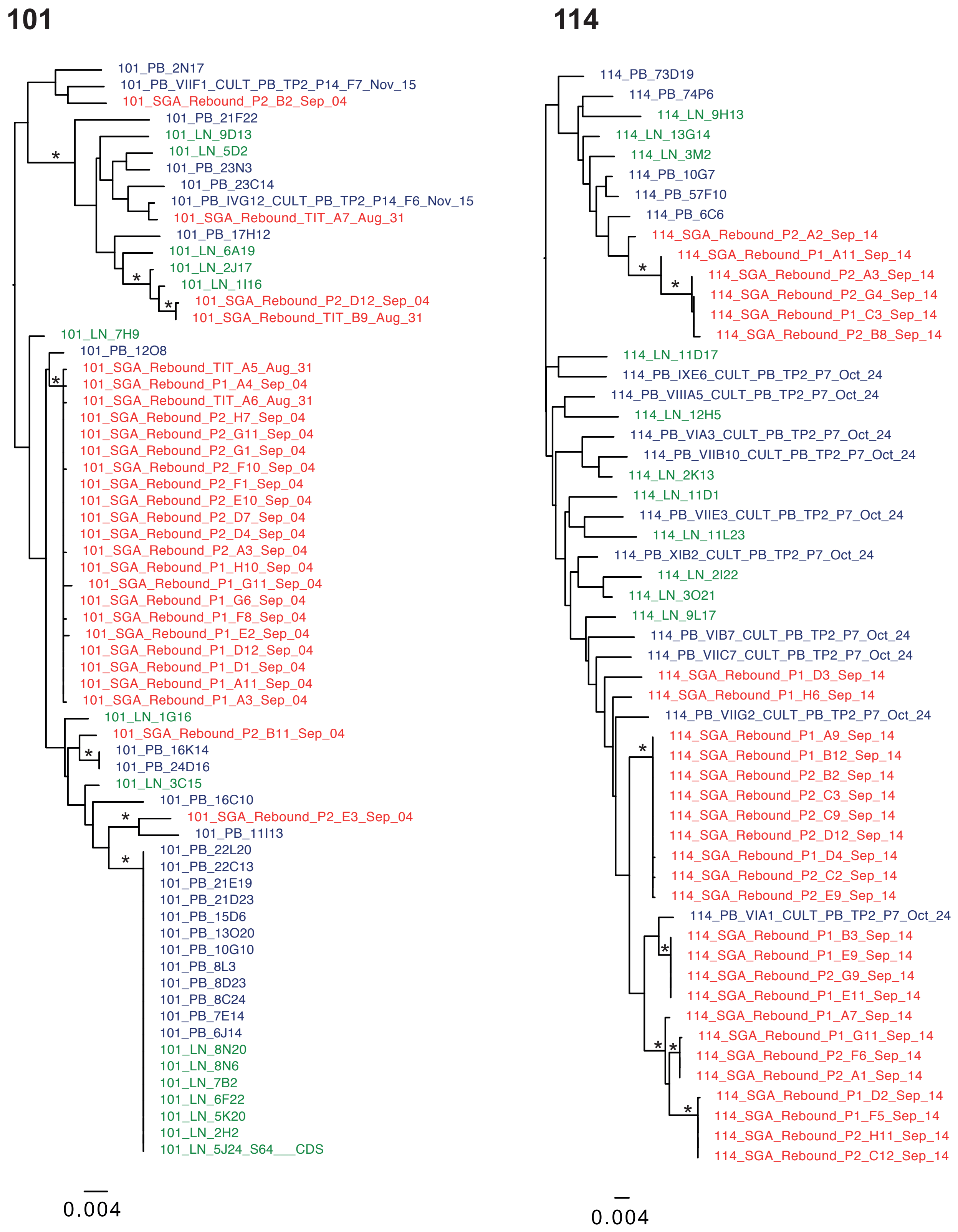

Extended Data Figure 3. Phylogenetic trees with sequence names. Maximum likelihood phylogenetic trees of env sequences from peripheral blood (PB) and lymph node (LN) intact near full-length sequences, PB viral outgrowth assay sequences and rebound SGA sequences. Asterisks indicate nodes with significant bootstrap values (bootstrap support ≥ 90%). The trees are midpoint rooted. Sequences from LN are red, sequences from blood are blue and rebound SGA sequences are red.

Extended Data Figure 3 (continued).

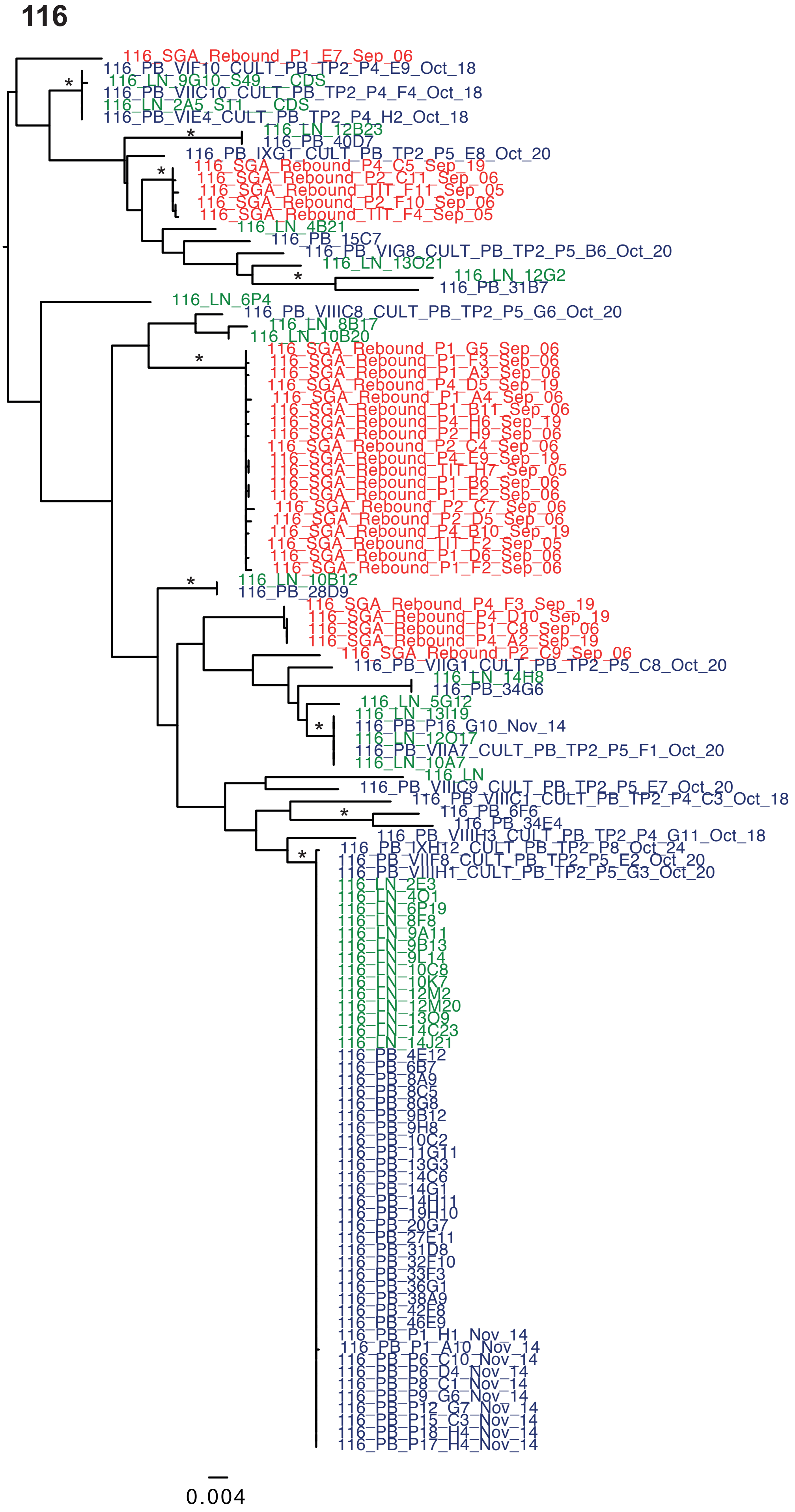

Extended Data Figure 3 (continued).

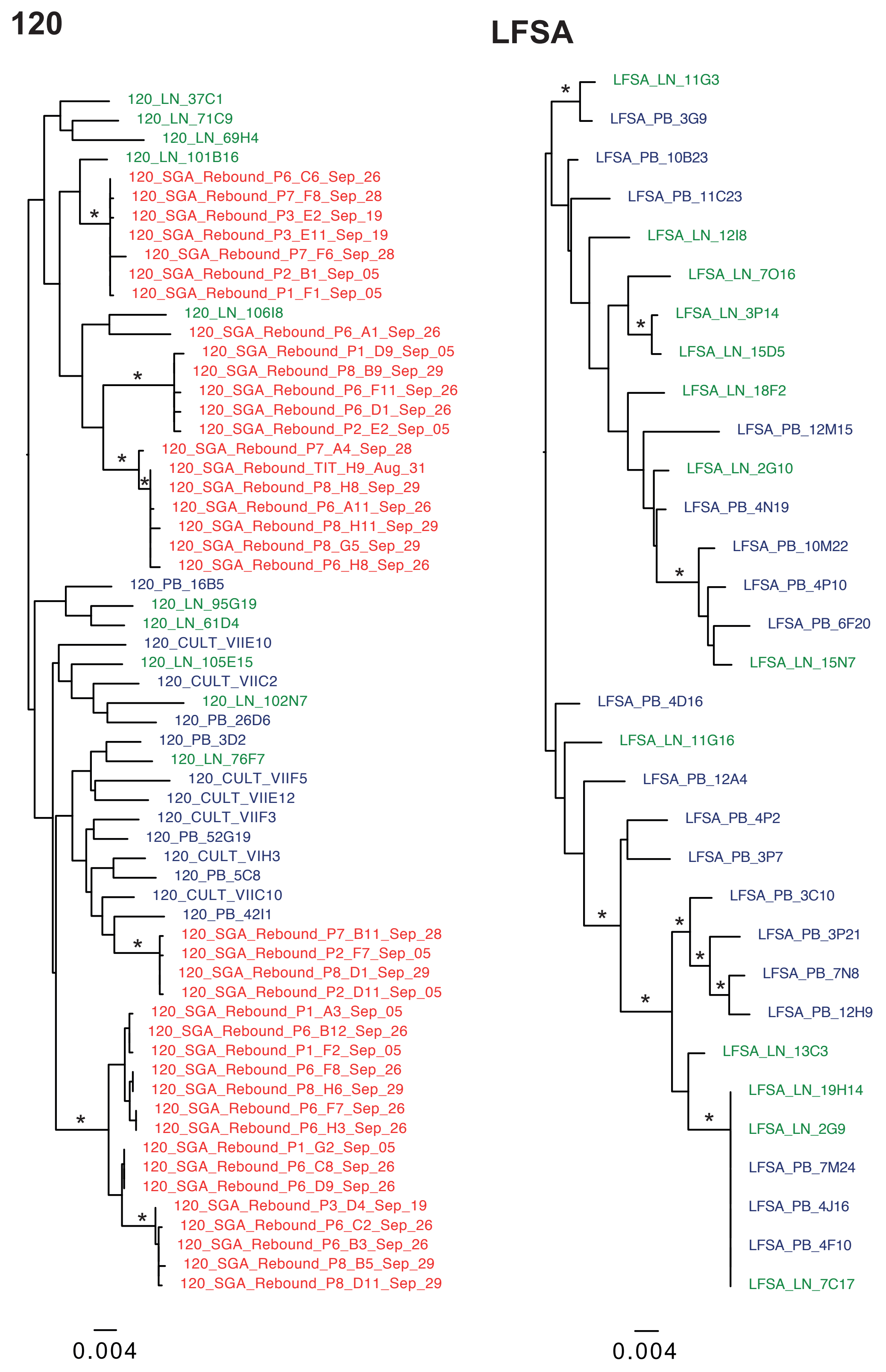

Supplement: Supplemental file 1 [file JVI.01920-18-s0001.pdf]
